# Age-Dependent Metastatic Spread and Survival: Cancer of Unknown Primary as a Model

**DOI:** 10.1038/srep23725

**Published:** 2016-03-24

**Authors:** Kari Hemminki, Nicholas Pavlidis, Konstantinos K. Tsilidis, Kristina Sundquist, Jianguang Ji

**Affiliations:** 1 Division of Molecular Genetic Epidemiology, German Cancer Research Center (DKFZ), Im Neuenheimer Feld 580, D-69120 Heidelberg, Germany; 2 Center for Primary Health Care Research, Lund University, 20502 Malmö, Sweden; 3 Department of Medical Oncology, School of Medicine, University of Ioannina, Ioannina, Greece; 4 Department of Hygiene and Epidemiology, University of Ioannina School of Medicine, University Campus, Ioannina 45110, Greece; 5 Department of Epidemiology and Biostatistics, The School of Public Health, Imperial College London, London W2 1PG, United Kingdom; 6 Stanford Prevention Research Center, Stanford University School of Medicine, Stanford, California 94305-5705, USA

## Abstract

In order to describe a novel approach for the clinical study of metastases, we provide here age-specific incidence and survival data for cancer of unknown primary (CUP). Metastases in various organs are found at CUP diagnosis, which have implications for prognosis, and we hypothesize similar prognostic implications for metastases found at diagnosis of primary cancers. We identified 33,224 CUP patients from the Swedish Cancer Registry and calculated incidence rates (IRs) for CUP development. Cox proportional hazards regression models were performed to estimate hazard ratios (HRs) for relative survival in CUP patients compared to the general population. In age-group specific analyses, a maximal IR was reached at age 85–89 years, followed by a marked decline to age 90+ (7-fold in men and 3-fold in women). The overall HR for relative survival declined systematically by age. CUP may be applied as an epidemiological age-incidence model for cancer metastases providing evidence in line with autopsy data that the metastatic potential, as shown by the incidence of CUP, appears to weaken markedly at age 85 years, depending on metastatic locations. The relative death rates were highest among young patients, which was probably entirely due to the low death rates in young background population.

Clinical epidemiology of cancer metastases has a shortage of available data because cancer registries record metastases only by the TNM classification, which states if any undefined metastasis can be found at diagnosis. Such data do not allow analysis of incidence or prognosis of patients with defined metastases. Cancer of unknown primary (CUP) may offer a novel model for clinical epidemiology of metastases because the diagnosis is based on metastases while no primary tumor can be found^1^,^2^,^3^. CUP is a fatal disease as are primary cancers with metastases; the median life-time for CUP is only 3 months^4^,^5^. Histology and site-specific location of metastases have prognostic implications and CUP with liver metastasis show the shortest survival of 2 months^4^,^6^. CUP is particularly attractive as a model because survival in CUP with metastases at a define location mimics, with few exceptions, survival times with primary cancers metastasized to identical locations^5^.

Survival in adult cancers depends on age at diagnosis, being most favorable in those diagnosed at young age^7^. However, the age-dependent differences in survival depend on the type of cancer; they are largest for fatal cancers, such as lung and stomach cancers, compared to more treatable cancers, such as breast cancer^7^. As most cancer deaths are due to metastases one could simplistically assume that metastases are more fatal among the elderly. However, the metastatic process is complex, including escape of malignant cells from the primary tumor and from immune surveillance, followed by seeding in diverse distant organ sites depending on tumor type^8^,^9^,^10^,^11^. Survival data in patients with metastasis have usually been compared between age groups without consideration of the higher background mortality in the elderly^12^,^13^,^14^. Moreover, at least for colorectal cancer, young patients present with more advance and aggressive diseases in spite of their better survival compared to the elderly^12^,^15^,^16^. A comprehensive analysis of cancer prevalence and mortality among centenarians showed a relative rarity of events in advanced age^17^.

In the present study we address in detail CUP risk by age at diagnosis according to histology and locations of metastases based on Swedish nation-wide data. Furthermore, survival in similar clinical categories is analyzed.

## Results

The number of CUP cases diagnosed between 1987 and 2012 was 33,224 with an overall IR of 12.3/100,000 person-years (Table 1). Adenocarcinoma (6.5) and undifferentiated carcinoma (2.0) had the highest IRs (per 100,000) among the histological subtypes of CUP. The most common designation of CUP location was ‘unspecified’ (IR 4.2), which commonly referred to cases with multiple metastatic sites. Abdomen and liver were the leading specific sites (2.6 and 2.2) where metastases were found, followed by thorax (1.1), neck (0.6) and bone (0.5). CUP incidence had slightly decreased from the period 1987–1999 (13.0) to 2000–2012 (11.6). Table 1 also shows hazard rates (HRs) for relative overall survival in patients diagnosed with CUP compared to those without CUP after adjustment for potential confounders. The overall HR was 15.5, highest for adenocarcinoma (19.1) and lowest for melanoma (6.7). CUP located in the liver conveyed the poorest survival (34.3) and CUP in the skin the best (6.2). The HR for the latter period was lower (18.2) compared to the former period (21.6), with non-overlapping 95% confidence intervals (95%CIs).

**Figure 1:**
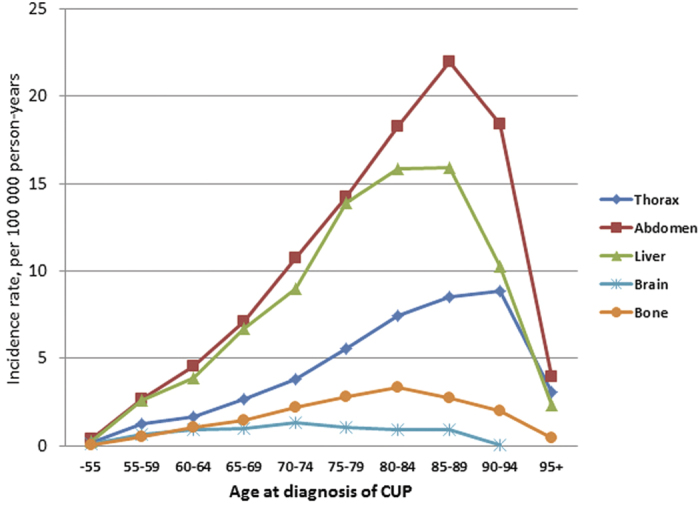
Incidence rate of CUP by location.

The age-specific incidence of CUP varied slightly between the metastatic sites (Fig. 1). There was a sharp maximum at age 85–89 years with an IR of 22.0 for abdominal metastatic sites, and for liver the maximum was reached between ages 80–89 years. The maximum incidence was reached at a slightly higher age for thoracic metastases, and at younger age for bone and brain metastases. A sharp decline in incidence of all metastatic sites was observed for age group 95+. Supplementary Table 1 shows the incidence data in detail for men (overall IR 10.6, maximal IR 76.0 at age 85–89) and women (overall IR 13.9, maximum 80.9 at age 85–89). The main reason for the higher incidence in women was the abdominal metastatic location (3.8 in women vs. 1.4 in men). This Table also shows that the male IR declined from 52.3 to 7.7 between age groups 90–94 and 95+, whereas in women the decline was from 66.3 to 21.3.

**Figure 2:**
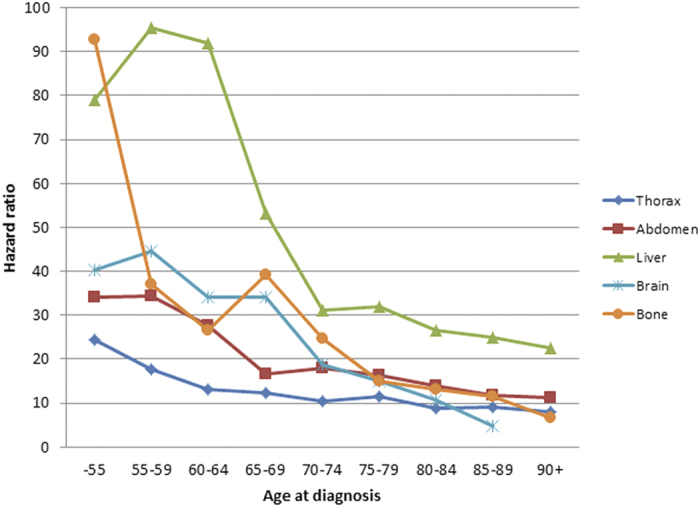
Hazard ratios of CUP by location.

Relative survival of CUP patients with site-specific metastases compared to the general Swedish population are shown in Fig. 2. The HR was high (close to 100) for patients with bone metastases at age <55 years, followed by a sharp decline at age 55–59 to a HR of <40, matching the HRs of patients with most other metastases. Patients with liver metastases showed high HRs (close to 100) up to age 65 years. Detailed HR data for men (overall HR 14.0) and women (15.5) are shown in Supplementary Table 2 with 95%CIs. The overall HR for both sexes declined systematically from the lowest to the highest age group. For both sexes adenocarcinoma was the most fatal histology and liver was the most fatal metastatic site. The survival improved moderately between 1987–1999 to after 2000 for men and women. Of note, the highest HR for men diagnosed before age 55 was found for patients with suprarenal metastases (108.2), whereas for women at this age bone metastases were most fatal (93.6).

**Figure 3:**
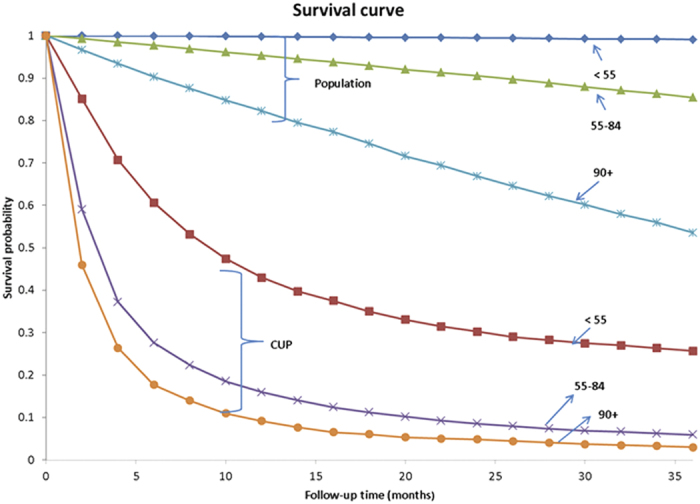
Kaplan Meier curves for CUP and for the background population (males and females combined) by age at diagnosis.

In order to put the above relative survival rates in perspective we show Kaplan Meier survival curves for the overall CUP survival (combined sexes) for three diagnostic age groups as well as for the population survival in the same age groups (Fig. 3). Although the survival in CUP diagnosed at <55 years was far better than in CUP diagnosed later (note the small difference between age groups 55–84 years and 90+ years), the young reference population also survived far better than the old ones. This explains the favorable HRs for relative survival in the elderly, in spite of their higher mortality rates.

## Discussion

The present study was based on 33,224 CUP patients from the nation-wide Swedish Cancer Registry. Because of large numbers most results were highly significant as shown by 95%CIs in the Tables. The incidence of most adult cancers is reaching a maximum at age 80 to 95 years followed by decline^17^,^18^. The peaking of incidence was an unsolvable problem to the early age-incidence models for cancer which assumed that the incidence increases as an exponent of age; thus, the peaking was initially discounted as biased statistics among the elderly^19^. However, the peaking was not a bias and many explanations have been offered to explain why cancer risk is not increased after a certain age, including the assumption that only a proportion of population is at risk of cancer^19^,^20^. The incidence data have been confirmed in autopsy studies, including a Japanese one covering 180,000 registered cases of which 3400 were over 90 years old^21^. In that study, the peak incidence of single cancers was in the 60 to 64 age group and that of multiple cancers was in the 80 to 84 age group. The prevalence of the different cancers peaked at the age of 75 year also in a in a large Swedish autopsy study^22^.

Metastases kill the patients but what is known about the age distribution? Many autopsy studies cited by Macieira-Coelho reported a decline in the rate of metastases in the elderly^23^. Similarly, the above Japanese autopsy study found the decline of metastases from 51% in age group 90–94 years to 38% in age group 100+years^21^. The present data show that CUP with the common metastatic locations, abdomen and liver, peaked at age 85 to 89 years, while CUP with thoracic metastases appeared somewhat later and CUP with bone and brain metastases appeared earlier. There was a remarkable drop in incidence from age group 85–89 to 90+, 7-fold in men and 3-fold in women.

The systematically decreasing HRs for CUP patients with increasing age suggest that the metastases lose aggressive character or, alternatively, that the host defense is better equipped to deal with them in advancing age. The data showed marked differences in relative survival depending on the site of metastases which have also been seen in an earlier study^4^. However the age-specific analysis showed that only patients with CUP metastases in the liver had poor survival throughout the age range while for patients with most other common metastases had only minor differences in HRs after age 70 years. Yet the overall HR remained at 14.0 for men and 8.9 for men at age 95+, reminding of the fatality of CUP. This is also obvious from the Kaplan Meier curves (Fig. 2).

Relative survival figures between two groups may differ because of the survival in cancer patients (nominator) or in the background population (denominator) differs. Thus in the assessment of survival among the young and old CUP patients one has to consider both parts of the equation. A crude assessment of ‘excess mortality’ (survival probability in CUP minus that of the population) based on Fig. 3 suggests that excess mortality is the reverse of the relative mortality and the elderly die earlier than young patients when deaths due to CUP are considered.

In summary the present study applies CUP as a model for cancer metastases, providing data on age-specific incidence and survival for histology- and site-specific metastases. These data in living humans are in line with autopsy data showing that the metastatic potential, as shown by the incidence in CUP, appears to weaken markedly at around 85 years, with some differences in metastatic locations. CUP remains a fatal disease at all ages and the relative death rates are highest among young patients because of their low death rate in the population; this may be the reverse in deaths related to CUP.

## Methods

This study included all individuals registered as residents of Sweden between 1987 and 2012. The data sources included the Swedish National Population and Housing Census, the Total Population Register, the Swedish Cancer Register, the Death of Cause Register, and the Immigration Register. These registers were provided to us by Statistics Sweden and the National Board of Health and Welfare. Patients diagnosed with CUP were identified from the Swedish Cancer Registry by the seventh International Classification of Diseases (ICD) codes of 199 from 1987 onwards. The Swedish Cancer Registry was founded in 1958 and has a national coverage and a completeness of over 90%^24^. It is maintained by the National Board of Health and Welfare. In Sweden, it is compulsory for clinicians and pathologists/cytologists to report all newly diagnosed cancers to the Cancer Registry. In addition, ICD-9 codes were also included in the Swedish Cancer Registry from 1987 onward, providing extra information about anatomic sites of CUP. ICD-9 codes of 1950 and 1960 were used to identify CUP located in neck; 1951, 1961, 1963, 1970–1973 to identify CUP in the thorax; 1952, 1953, 1962, 1965, 1974–1976, 1979–1981, 1986 to identify CUP in the abdomen; 1977 to identify CUP in the liver; 1982 to identify CUP in the skin; 1983 and 1984 to identify CUP in the brain; 1985 to identify CUP in the bone; 1989 to identify CUP in the suprarenal; 1990 and 1991 to identify CUP unspecified. Pathology-anatomy diagnosis (PAD) codes were used (WHO/HS/CANC/24.1) to identify the histology of CUP. PAD 096 were used to identify adenocarcinoma; 146 for squamous cell carcinoma; 176 for malignant melanoma; 196 for undifferentiated tumor. For CUP histology, 25% of the patients had a missing or other histology.

Additional linkages were made to the Swedish National Population and Housing Census^25^ to obtain information on individual-level characteristics, such as year of birth, and country of birth; to the Cause of Death Register to identify date of death; and to the Emigration Registry to identify date of emigration. All linkages were performed using individual national identification numbers, which were replaced with serial numbers in order to preserve anonymity. The incidence rate (IR) of CUP was calculated as the number of newly diagnosed cancer cases divided by the person-years during the follow-up. IRs were calculated separately by histological types, metastatic locations, age and period at diagnosis.

Each CUP patient was matched with up to five persons from the general population without a diagnosis of CUP according to gender and date of birth. Cox proportional hazards regression models were used to estimate hazard rates (HRs) for relative overall survival in patients diagnosed with CUP compared to those without CUP after adjustment for potential confounding by age (continuous), education level, and geographic region of residence. We censored individuals (that is, treated them as no longer under observation or at risk of the study outcome) at the time of death from any cause, at the end of the follow-up period (December 31, 2012), or at the time of emigration. The proportional hazards assumption was tested by plotting the log of the negative log of the survival function versus the log of time; these were parallel in line with the proportional hazards assumption. All analyses were performed using SAS version 9.2 (SAS Institute, Cary, NC, USA).

### Ethical statement

The study was approved by the Ethical Committee of Lund University and the study was conducted in accordance with the approved guidelines.

## Additional Information

**How to cite this article**: Hemminki, K. *et al*. Age-Dependent Metastatic Spread and Survival: Cancer of Unknown Primary as a Model. *Sci. Rep*. **6**, 23725; doi: 10.1038/srep23725 (2016).

## Supplementary Material

Supplementary Information

## Figures and Tables

**Table 1:** Incidence rate (per 100,000 person-years) and hazard ratios among patients with cancer of unknown primary in Sweden.

Subtype	No.	IR	HR	95%CI	
Total	33,224	12.3	15.5	15.2	15.8
Histology					
Adenocarcinoma	30,392	6.5	19.1	18.6	19.6
SCC	1,584	0.6	8.1	7.3	9.1
Melanoma	1,360	0.5	6.7	5.9	7.6
Undifferentiated	5,316	2.0	16.8	16.0	17.7
Location					
Neck	1,630	0.6	8.6	7.7	9.5
Thorax	2,873	1.1	9.8	9.1	10.5
Abdomen	7,061	2.6	16.3	15.7	16.9
Liver	5,993	2.2	34.3	32.4	36.3
Skin	499	0.2	6.2	5.3	7.3
Brain	817	0.3	22.7	19.6	26.3
Bone	1,336	0.5	20.9	18.6	23.5
Suprarenal	564	0.2	11.2	9.6	13.0
Unspecified	11,312	4.2	16.3	15.7	16.9
Diagnosis period					
1987–1999	17,390	13.0	21.6	20.9	22.3
2000–2012	15,834	11.6	18.2	17.6	18.7
