# Supplementary material for: Age-Dependent Metastatic Spread and Survival: Cancer of Unknown Primary as a Model
Source: Sci Rep. 2016 Mar 24;6:23725. doi: 10.1038/srep23725 (PMC4806321; doi:10.1038/srep23725)
Supplement: Supplementary Information [file srep23725-s1.pdf]

AGE-DEPENDENT METASTATIC SPREAD AND SURVIVAL: CANCER OF UNKNOWN PRIMARY AS A MODEL

Kari Hemminki, [1,2], Nicholas Pavlidis [3], Konstantinos K. Tsilidis[4,5], Kristina Sundquist, [2,6], Jianguang Ji [2]

SUPPLEMENTARY TABLES 1 and 2.

Supplementary Table 1. Incidence rate of cancer of unknown primary in Sweden by gender

Subtype	<55		55-69		70-84		85-89		90-94		95+		All	
	**No.**	**IR**	**No.**	**IR**	**No.**	**IR**	**No.**	**IR**	**No.**	**IR**	**No.**	**IR**	**No.**	**IR**
**Men**														
Total	1,525	1.5	4,680	22.5	6,747	60.9	1,056	75.9	286	52.3	55	7.7	14,349	10.6
Histology														
Adenocarcinoma	593	0.6	2,263	10.8	3,200	28.8	450	32.0	111	20.3	17	2.6	6,634	4.9
SCC	147	0.1	381	1.8	366	3.3	49	3.4	16	2.9	5	0.7	964	0.7
Melanoma	207	0.2	290	1.4	250	2.3	31	2.3	11	2.0	6	0.7	795	0.6
Undifferentiated	257	0.3	848	4.1	1,192	10.7	159	11.6	36	6.6	9	1.1	2,501	1.9
Location														
Neck	187	0.2	347	2.0	357	3.7	50	4.1	20	4.4	1	0.2	962	0.7
Thorax	145	0.2	402	2.3	652	6.8	124	10.3	32	7.1	8	1.6	1,363	1.0
Abdomen	234	0.3	606	3.4	850	8.8	135	11.3	42	9.3	3	0.5	1,870	1.4
Liver	189	0.2	909	5.2	1,420	14.7	225	18.8	52	11.5	8	1.2	2,803	2.1
Skin	17	0.0	61	0.3	102	1.1	18	1.5	11	2.4	4	0.7	213	0.2
Brain	68	0.1	182	1.0	161	1.6	13	1.2	1	0.1	0		425	0.3
Bone	61	0.1	232	1.3	361	3.7	42	3.6	13	2.9	2	0.2	711	0.5
Suprarenal	15	0.0	82	0.5	126	1.3	23	1.9	5	1.1	1	0.2	252	0.2
Unspecified	553	0.7	1,691	9.7	2,476	25.7	380	31.3	101	22.3	25	4.7	5,226	3.9
Diagnosis period														
1987-1999	843	1.7	2,419	26.9	3,871	72.4	526	89.9	140	65.2	20	8.5	7,819	11.7
2000-	682	1.7	2,261	23.2	2,876	61.4	530	81.5	146	56.5	35	9.8	6,530	9.6
**Women**														
Total	1612	1.7	5025	24.6	9081	65.8	2023	80.9	757	66.3	161	21.3	18,659	13.9
Histology														
Adenocarcinoma	819	0.9	3,120	15.2	5,474	39.6	1,094	43.7	369	32.3	78	9.9	10,954	8.2
SCC	97	0.1	213	1.0	233	1.7	46	1.9	16	1.4	5	0.8	610	0.5
Melanoma	160	0.2	173	0.8	169	1.2	32	1.3	17	1.5	4	0.6	555	0.4
Undifferentiated	270	0.3	774	3.8	1,380	9.9	272	10.8	78	6.8	16	2.1	2,790	2.1
Location														
Neck	110	0.1	214	1.2	254	2.0	48	2.0	33	3.2	4	0.8	663	0.5
Thorax	150	0.2	350	2.0	679	5.4	176	7.6	101	9.7	23	4.3	1,479	1.1
Abdomen	353	0.5	1,294	7.4	2,582	20.7	639	27.5	234	22.4	43	6.9	5,145	3.8
Liver	192	0.3	840	4.8	1,672	13.4	330	14.4	102	9.8	23	3.4	3,159	2.4
Skin	28	0.0	74	0.4	128	1.0	33	1.4	15	1.4	2	0.4	280	0.2
Brain	82	0.1	167	1.0	125	1.0	17	0.7	1	0.1	0		392	0.3
Bone	51	0.1	183	1.0	313	2.5	49	2.2	17	1.6	5	0.6	618	0.5
Suprarenal	34	0.0	89	0.5	134	1.1	31	1.3	17	1.6	3	0.4	308	0.2
Unspecified	540	0.7	1,667	9.5	2,889	23.1	628	27.1	223	21.3	57	10.5	6,004	4.5
Diagnosis period														
1987-1999	872	1.8	2,442	26.8	4,734	68.6	995	88.7	348	74.4	80	35.4	9,471	14.2
2000-	740	1.9	2,583	27.1	4,347	73.0	1,028	83.4	409	67.9	81	20.4	9,188	13.6

Supplementary Table 2. Relative survival in patients with CUP by gender

Subtype	<55					55-69					70-84					85-89					90+					All				
	No.	Death	HR	95%CI		No.	Death	HR	95%CI		No.	Death	HR	95%CI		No.	Death	HR	95%CI		No.	Death	HR	95%CI		No.	Death	HR	95%CI	
**Men**																														
Total	1,525	1200	25.5	23.0	28.3	4,680	4336	18.7	17.9	19.5	6,747	6620	12.9	12.5	13.3	1,056	1041	10.9	10.1	11.9	341	339	8.9	7.7	10.2	14,349	13,536	14.0	13.7	14.4
Histology																														
Adenocarcinoma	593	560	51.8	43.7	61.3	2,263	2203	30.2	28.2	32.3	3,200	3182	21.0	19.9	22.1	450	448	17.0	14.8	19.4	128	127	9.3	7.4	11.7	6,634	6,520	23.1	22.2	23.9
SCC	147	60	9.1	6.2	13.4	381	258	7.2	6.2	8.5	366	337	5.4	4.7	6.1	49	42	3.9	2.7	5.7	21	21	14.7	7.3	29.8	964	718	6.0	5.4	6.5
Melanoma	207	141	23.2	16.9	31.9	290	229	9.5	7.9	11.4	250	215	4.2	3.6	4.9	31	29	3.2	2.1	4.9	17	17	4.5	2.4	8.3	795	631	6.7	6.1	7.4
Undifferentiated	257	220	30.7	23.9	39.4	848	803	26.3	23.5	29.3	1,192	1173	15.0	13.9	16.3	159	157	10.4	8.5	12.8	45	44	5.6	3.9	8.0	2,501	2,397	15.8	14.9	16.7
Location																														
Neck	187	83	11.1	7.8	15.7	347	239	7.6	6.5	9.0	357	333	5.7	5.0	6.4	50	45	4.1	2.9	5.8	21	21	16.5	8.0	34.1	962	721	6.3	5.7	6.9
Thorax	145	110	19.0	13.9	26.0	402	373	16.1	14.0	18.5	652	639	12.7	11.5	14.1	124	122	8.2	6.5	10.3	40	40	7.0	4.8	10.4	1,363	1,284	12.6	11.7	13.6
Abdomen	234	184	33.3	25.1	44.2	606	564	18.0	16.0	20.3	850	841	14.7	13.4	16.1	135	134	12.2	9.7	15.2	45	45	13.9	9.2	20.9	1,870	1,768	15.7	14.7	16.7
Liver	189	183	80.5	57.6	112.6	909	894	63.6	55.6	72.8	1,420	1418	30.1	27.7	32.8	225	224	24.6	19.8	30.5	60	60	24.2	15.7	37.3	2,803	2,779	35.0	32.9	37.3
Skin	17	13	20.7	8.1	53.1	61	55	13.3	9.1	19.6	102	94	7.3	5.7	9.4	18	18	3.3	1.9	5.8	15	15	7.0	3.6	13.9	213	195	7.5	6.3	9.0
Brain	68	62	29.5	18.6	46.7	182	178	30.6	23.9	39.3	161	161	26.9	21.0	34.4	13	13	33.2	11.7	94.0	1	1				425	415	25.8	22.2	30.0
Bone	61	59	59.6	35.2	101.1	232	229	30.6	24.9	37.8	361	357	14.9	12.9	17.3	42	42	16.1	10.1	25.8	15	14	4.3	2.3	8.2	711	701	15.5	14.0	17.1
Suprarenal	15	12	108.2	22.4	521.7	82	77	14.4	10.4	19.8	126	117	6.0	4.8	7.4	23	23	11.3	6.3	20.3	6	6	13.4	3.6	49.4	252	235	8.2	7.0	9.7
Unspecified	553	451	26.8	22.6	31.8	1,691	1580	19.8	18.4	21.3	2,476	2424	12.6	12.0	13.3	380	374	11.1	9.7	12.7	126	125	8.3	6.6	10.4	5,226	4,954	14.2	13.6	14.7
Diagnosis period																														
1987-1999	843	630	69.4	55.6	86.8	2,419	2171	31.4	29.1	33.8	3,871	3655	16.4	15.7	17.2	526	511	15.4	13.6	17.4	160	153	14.9	11.9	18.7	7,819	7,120	19.7	19.0	20.4
2000-	682	488	50.8	40.2	64.3	2,261	1972	26.4	24.4	28.5	2,876	2757	14.5	13.8	15.3	530	515	9.1	8.1	10.2	181	180	7.2	6.0	8.7	6,530	5,912	15.5	14.9	16.0
**Women**																														
Total	1612	1322	38.8	34.7	43.4	5025	4616	21.6	20.7	22.6	9081	8869	15.2	14.7	15.6	2023	2007	11.6	11.0	12.3	918	912	9.0	8.3	9.8	18,659	17,726	15.5	15.2	15.8
Histology																														
Adenocarcinoma	819	728	44.1	38.0	51.2	3,120	2951	29.0	27.3	30.7	5,474	5368	18.7	18.0	19.4	1,094	1085	13.5	12.4	14.6	447	445	8.6	7.7	9.7	10,954	10,577	19.1	18.6	19.6
SCC	97	54	25.9	15.0	44.7	213	144	9.3	7.4	11.7	233	221	7.6	6.5	8.9	46	46	6.9	4.9	9.8	21	21	7.2	4.2	12.5	610	486	8.1	7.3	9.1
Melanoma	160	112	36.4	23.6	56.1	173	125	9.7	7.5	12.5	169	137	4.4	3.6	5.4	32	29	4.5	2.9	7.0	21	20	4.6	2.6	8.0	555	423	6.7	5.9	7.6
Undifferentiated	270	231	59.6	44.1	80.5	774	725	22.5	20.1	25.2	1,380	1352	16.5	15.3	17.7	272	271	12.8	10.9	15.0	94	94	8.2	6.4	10.6	2,790	2,673	16.8	16.0	17.7
Location																														
Neck	110	68	23.8	14.8	38.1	214	165	11.8	9.4	14.7	254	242	7.8	6.7	9.1	48	47	7.0	4.8	10.1	37	37	6.8	4.6	10.1	663	559	8.6	7.7	9.5
Thorax	150	112	33.7	23.1	49.2	350	295	12.2	10.4	14.3	679	656	8.6	7.8	9.5	176	176	10.3	8.5	12.4	124	124	8.6	6.9	10.8	1,479	1,363	9.8	9.1	10.5
Abdomen	353	287	35.1	27.8	44.3	1,294	1183	24.5	22.4	26.9	2,582	2510	16.5	15.6	17.4	639	633	11.9	10.7	13.1	277	276	10.8	9.2	12.6	5,145	4,889	16.3	15.7	16.9
Liver	192	180	93.6	65.1	134.6	840	829	77.1	66.7	89.2	1,672	1656	29.4	27.3	31.8	330	330	25.8	21.8	30.5	125	125	21.7	16.3	28.9	3,159	3,120	34.3	32.4	36.3
Skin	28	22	51.3	17.4	151.4	74	54	7.8	5.4	11.1	128	116	6.7	5.3	8.3	33	32	4.9	3.2	7.4	17	17	3.8	2.1	6.6	280	241	6.2	5.3	7.3
Brain	82	78	57.7	35.3	94.3	167	163	56.1	41.9	75.3	125	123	15.4	12.2	19.6	17	17	3.5	2.0	6.1	1	1				392	382	22.7	19.6	26.3
Bone	51	49	93.6	65.1	134.6	183	175	31.4	24.4	40.3	313	310	19.1	16.3	22.4	49	49	10.7	7.4	15.6	22	22	15.2	8.2	28.3	618	605	20.9	18.6	23.5
Suprarenal	34	27	72.2	25.9	201.4	89	79	14.7	10.7	20.4	134	127	13.4	10.6	17.0	31	30	7.1	4.5	11.0	20	19	3.9	2.3	6.6	308	282	11.2	9.6	13.0
Unspecified	540	446	38.6	31.9	46.8	1,667	1539	21.7	20.1	23.5	2,889	2833	16.8	15.9	17.7	628	622	12.3	11.1	13.6	280	276	8.1	7.0	9.4	6,004	5,716	16.3	15.7	16.9
Diagnosis period																														
1987-1999	872	674	99.3	77.4	127.5	2,442	2114	41.0	37.7	44.7	4,734	4420	22.0	21.0	23.0	995	958	12.6	11.6	13.7	428	413	10.4	9.2	11.8	9,471	8,579	21.6	20.9	22.3
2000-	740	546	74.3	57.6	96.0	2,583	2285	39.0	35.9	42.3	4,347	4156	17.8	17.0	18.6	1,028	1013	13.5	12.4	14.7	490	484	8.7	7.8	9.8	9,188	8,484	18.2	17.6	18.7
